# Comparison of Severity of Illness Scores and Artificial Intelligence Models That Are Predictive of Intensive Care Unit Mortality: Meta-analysis and Review of the Literature

**DOI:** 10.2196/35293

**Published:** 2022-05-31

**Authors:** Cristina Barboi, Andreas Tzavelis, Lutfiyya NaQiyba Muhammad

**Affiliations:** 1 Indiana University Purdue University Regenstrief Institue Indianapolis, IN United States; 2 Medical Scientist Training Program Feinberg School of Medicine Chicago, IL United States; 3 Department of Biomedical Engineering Northwestern University Chicago, IL United States; 4 Department of Preventive Medicine and Biostatistics Northwestern University Evanston, IL United States

**Keywords:** artificial intelligence, machine learning, intensive care unit mortality, severity of illness models

## Abstract

**Background:**

Severity of illness scores—Acute Physiology and Chronic Health Evaluation, Simplified Acute Physiology Score, and Sequential Organ Failure Assessment—are current risk stratification and mortality prediction tools used in intensive care units (ICUs) worldwide. Developers of artificial intelligence or machine learning (ML) models predictive of ICU mortality use the severity of illness scores as a reference point when reporting the performance of these computational constructs.

**Objective:**

This study aimed to perform a literature review and meta-analysis of articles that compared binary classification ML models with the severity of illness scores that predict ICU mortality and determine which models have superior performance. This review intends to provide actionable guidance to clinicians on the performance and validity of ML models in supporting clinical decision-making compared with the severity of illness score models.

**Methods:**

Between December 15 and 18, 2020, we conducted a systematic search of PubMed, Scopus, Embase, and IEEE databases and reviewed studies published between 2000 and 2020 that compared the performance of binary ML models predictive of ICU mortality with the performance of severity of illness score models on the same data sets. We assessed the studies' characteristics, synthesized the results, meta-analyzed the discriminative performance of the ML and severity of illness score models, and performed tests of heterogeneity within and among studies.

**Results:**

We screened 461 abstracts, of which we assessed the full text of 66 (14.3%) articles. We included in the review 20 (4.3%) studies that developed 47 ML models based on 7 types of algorithms and compared them with 3 types of the severity of illness score models. Of the 20 studies, 4 (20%) were found to have a low risk of bias and applicability in model development, 7 (35%) performed external validation, 9 (45%) reported on calibration, 12 (60%) reported on classification measures, and 4 (20%) addressed explainability. The discriminative performance of the ML-based models, which was reported as AUROC, ranged between 0.728 and 0.99 and between 0.58 and 0.86 for the severity of illness score–based models. We noted substantial heterogeneity among the reported models and considerable variation among the AUROC estimates for both ML and severity of illness score model types.

**Conclusions:**

ML-based models can accurately predict ICU mortality as an alternative to traditional scoring models. Although the range of performance of the ML models is superior to that of the severity of illness score models, the results cannot be generalized due to the high degree of heterogeneity. When presented with the option of choosing between severity of illness score or ML models for decision support, clinicians should select models that have been externally validated, tested in the practice environment, and updated to the patient population and practice environment.

**Trial Registration:**

PROSPERO CRD42021203871; https://tinyurl.com/28v2nch8

## Introduction

### Background

In the United States, intensive care unit (ICU) care costs account for 1% of the US gross domestic product, underscoring the need to optimize its use to attenuate the continued increase in health care expenditures [1]. Models that characterize the severity of illnesses of patients who are critically ill by predicting complications and ICU mortality risk can guide organizational resource management and planning, implementation and support of critical clinical protocols, and benchmarking and are proxies for resource allocation and clinical performance [2]. Although the medical community values the information provided by such models, they are not consistently used in practice because of their complexity, marginal predictive capacity, and limited internal or external validation [2-5].

Severity of illness score models require periodic updates and customizations to reflect changes in medical care and regional case pathology [6]. Scoring models are prone to high interrater variability, are less accurate for patients with increased severity of illness score or specific clinical subgroups, are not designed for repeated applications, and cannot represent patients’ status trends [7]. The Acute Physiology and Chronic Health Evaluation (APACHE)-II (APACHE-II) and Simplified Acute Physiology Score (SAPS), developed in the 80s, are still in use [8]. The underlying algorithms for APACHE-IV are in the public domain and are available at no cost; however, their use is time intensive and is facilitated by software that requires payments for licensing implementation and maintenance [9]. Compared with SAPS-III, which uses data exclusively obtained within the first hour of ICU admission [10], APACHE-IV uses data from the first day (24 hours) [11]. Although the Sequential Organ Failure Assessment (SOFA) is an organ dysfunction score that detects differences in the severity of illness and is not designed to predict mortality, it is currently used to estimate mortality risk based on the mean, highest, and time changes accrued in the score during the ICU stay [11].

The availability of machine-readable data from electronic health records enables the analysis of large volumes of medical data using machine learning (ML) methods. ML algorithms enable the exploration of high-dimensional data and the extraction of features to develop models that solve classification or regression problems. These algorithms can fit linear and nonlinear associations and interactions between predictive variables and relate all or some of the predictive variables to an outcome. The increased flexibility of ML models comes with the risk of overfitting training data; therefore, model testing on external data is essential to ensure adequate performance on previously unseen data. In model development, the balance between the model’s accuracy and generalizability, or bias and variance, is achieved through model training on a *training set* and hyperparameter optimization on a *tuning set*. Once a few models have been trained, they can be internally validated on a *split-sample* data set or cross-validated; the candidate model chosen is then validated on an unseen *test data set* to calculate its performance metrics and out of sample error [12]. The choice of algorithm is critical for providing a balance between interpretability, accuracy, and susceptibility to bias and variance [13]. Compared with the severity of illness scores, ML models can incorporate large numbers of covariates and temporal data, nonlinear predictors, trends in measured variables, and complex interactions between variables [14]. Numerous ML algorithms have been integrated into ICU predictive models, such as artificial neural networks (NNs), deep reinforcement learning, support vector machines (SVMs), random forest models, genetic algorithms, clinical trajectory models, gradient boosting models, k-nearest neighbor, naive Bayes, and the Ensemble approach [15]. Despite the rapidly growing interest in using ML methods to support clinical care, modeling processes and data sources have been inadequately described [16,17]. Consequently, the ability to validate and generalize the current literature’s results is questionable.

### Objectives

This study aims to systematically review and meta-analyze studies that compare binary classification ML models with the severity of illness scores for predicting ICU mortality and determine which models have superior performance. This review intends to provide actionable guidance to clinicians on the prognostic value of ML models compared with the severity of illness scores in supporting clinical decision-making, as well as on their performance, in the context of the current guidelines [18] and recommendations for reporting ML analysis in clinical research [19] (Table 1).

**Table 1:** Recommended structure for reporting ML^a^ models.

Research question and ML justification	Data sources and preprocessing (feature selection)	Model training and validation
Clinical question	Population	Hardware, software, and packages used
Intended use of the result	Sample record and measurement characteristics	Evaluation (calibration and discrimination)
Defined problem type	Data collection and quality	Configuration (parameters and hyperparameters)
Available data	Data structure and types	Model optimization and generalization (hyperparameter tuning and parameter limits)
Defined ML method and rationale	Differences between evaluation and validation sets	Validation method and data split and cross-validation
Defined evaluation measures, training protocols, and validation	Data preprocessing (data aggregation, missing data, transformation, and label source)	Validation method performance metrics on an external data set
N/A^b^	Input configuration	Reproducibility, code reuse, and explainability

^a^ML: machine learning.

^b^N/A: not applicable.

## Methods

We conducted a systematic review of the relevant literature. The research methods and reporting followed the PRISMA (Preferred Reporting Items for Systematic Reviews and Meta-Analyses) 2020 statement and guide to review and meta-analysis of prediction models [20,21].

### Information Sources and Search Strategy

Between December 15 and 18, 2020, we performed a comprehensive search in the bibliographic databases PubMed, Scopus, Embase, and IEEE of the literature published between December 2000 and December 15, 2020. These databases were available free of charge from the university library. We selected PubMed for its significance in biomedical electronic research; Scopus for its wide journal range, keyword search, and citation analysis; Embase because of its European union literature coverage; and IEEE Xplore for its access to engineering and computer science literature.

The search terms included control terms (Medical Subject Headings and Emtree) and free-text terms. The filters applied during the search of all 4 databases were *Humans* and *Age:* *Adult*. A search of the PubMed database using the terms (*AI artificial intelligence*) OR (*machine learning*) AND (*intensive care unit*) AND (*mortality)* identified 125 articles. The Scopus database was searched using the terms KEY (*machine learning*) OR KEY (*artificial-intelligence*) AND KEY (*intensive care unit*) AND KEY (*mortality*) revealed 182 articles. The Embase database queries using the terms (*AI Artificial Intelligence*) OR (*machine learning*) AND (*intensive care unit*) AND (*mortality*) resulted in 103 articles. The IEEE database search using the terms (*machine learning*) OR (*artificial intelligence*) AND (*intensive care unit*) AND (*mortality*) produced 51 citations.

A total of 2 authors (CB and AT) screened titles and abstracts and recorded the reasons for exclusion. The same authors (CB and AT) independently reviewed the previously selected full-text articles to determine their eligibility for quantitative and qualitative assessments. Both authors revisited the discrepancies to guarantee database accuracy and checked the references of the identified articles for additional papers. A third researcher (LNM) was available to resolve any disagreements.

### Eligibility Criteria and Study Selection

We included studies that compared the predictive performance of newly developed ML classification models predictive of ICU mortality with the severity of illness score models on the same data sets in the adult population. To be included in the review, the studies had to provide information on the patient cohort, model development and validation, and performance metrics. Both prospective and retrospective studies were eligible for inclusion.

### Data Collection Process

Data extraction was performed by CB, reviewed by AT, and guided by the CHARMS (Critical Appraisal and Data Extraction for Systematic Reviews of Prediction Modeling Studies) checklist [22] specifically designed for systematic reviews of prognostic prediction models. The methodological qualities of the included studies were appraised with guidance from the Prediction model Risk of Bias (ROB) Assessment Tool (PROBAST) [23]. The reported features of the ML models are shown in Table 2.

**Table 2:** CHARMS (Checklist for Critical Appraisal and Data Extraction for Systematic Reviews of Prediction Modeling Studies) checklist.

Author	Data source (description)	Outcome mortality	Data preparation		Model training				Predictive performance					Generalizability				
			A^a^	B^b^	C^c^	D^d^	E^e^	F^f^		G^g^	H^h^	I^i^	J^j^		K^k^	L^l^	M^m^	N^n^
Pirracchio et al [1]	MIMIC^o^ 2	Hospital				✓	✓	✓		✓			✓			✓		✓
Nielsen et al [24]	Danish ICU^p^	Hospital 30/90 days	✓	✓	✓	✓	✓			✓	✓		✓				✓	
Nimgaonkar et al [25]	ICU India	Hospital				✓	✓	✓		✓								
Xia et al [26]	MIMIC 3	28 days/hospital	✓	✓		✓	✓			✓	✓						✓	✓
Purushotham et al [27]	MIMIC 3	Hospital, 2 days, 3 days, 30 days, 1 year	✓	✓		✓	✓			✓	✓		✓				✓	✓
Nanayakkara et al [28]	ANZICS^q^	Hospital	✓	✓	✓	✓	✓	✓		✓	✓				✓	✓	✓	✓
Meyer et al [29]	Germany	Hospital		✓		✓	✓			✓	✓		✓			✓		✓
Meiring et al [7]	CCHIC^r^ United Kingdom	Hospital	✓	✓	✓	✓	✓			✓							✓	✓
Lin et al [30]	MIMIC 3	Hospital	✓	✓		✓	✓	✓		✓	✓							
Krishnan et al [31]	MIMIC 3	ICU	✓	✓		✓	✓			✓	✓					✓		
Kang et al [32]	Korea	Hospital	✓		✓	✓	✓	✓		✓	✓				✓			
Johnson et al [33]	United Kingdom	ICU and hospital	✓	✓		✓	✓	✓		✓		✓	✓			✓		
Holmgren et al [34]	Sweden	Hospital and 30 days			✓	✓	✓	✓		✓	✓						✓	✓
Garcia-Gallo et al [35]	MIMIC 3	Hospital and 1 year	✓	✓	✓	✓	✓	✓		✓	✓						✓	✓
El-Rashidy et al [36]	MIMIC 3	ICU and hospital	✓	✓		✓	✓			✓	✓		✓				✓	✓
Silva et al [37]	EURICUS^s^ 2	ICU	✓	✓	✓	✓	✓			✓	✓	✓			✓	✓		
Caicedo-Torres et al [38]	MIMIC 3	ICU	✓	✓		✓	✓			✓	✓				✓			
Deshmukh et al [39]	eICU-CRD^t^	ICU	✓	✓	✓	✓	✓			✓	✓				✓		✓	
Ryan et al [40]	MIMIC 2	ICU and hospital	✓	✓	✓	✓	✓			✓	✓		✓				✓	✓
Mayaud et al [41]	MIMIC 2	Hospital	✓	✓		✓	✓	✓		✓		✓						

^a^Data normalization/outlier addressed.

^b^Missing data addressed.

^c^Hyperparameter optimization addressed.

^d^Overfitting/shrinkage and cross-validation addressed.

^e^Predictor selection, full model versus backward elimination.

^f^Calibration assessed (Brier, Hosmer-Lemeshow, and calibration plot).

^g^Discrimination/reclassification performed (net reclassification improvement/integrated discrimination improvement).

^h^Classification reported.

^i^Recalibration performed.

^j^Externally validated.

^k^Explainability addressed/decision curve analysis.

^l^Clinical applicability addressed.

^m^Prediction span defined.

^n^Intended moment of use reported.

^o^MIMIC: Medical Information Mart for Intensive Care.

^p^ICU: intensive care unit.

^q^ANZICS: Australia New Zealand Intensive Care Unit Society.

^r^CCHIC: Critical Care Health Informatics Collaborative.

^s^EURICUS: European ICU studies.

^t^eICU CRD: Electronic ICU Collaborative Research Database.

### Assessment of the ROB and Quality of Reviewed Studies

The reviewers used the PROBAST tool to assess the methodological quality of each study for ROB and concerns regarding applicability in 4 domains: study participants, predictors, outcome, and analysis [23]. The reviewers evaluated the applicability of the selected studies by assessing the extent to which the studied outcomes matched the goals of the review in the 4 domains. We evaluated the ROB by assessing the primary study design and conduct, predictor selection process, outcome definition, and performance analysis. The ROB in the reporting models’ performance was appraised by exploring the reported measures of calibration (model’s predicted risk of mortality vs the observed risk), discrimination (model’s ability to discriminate between patients who are alive or expired), classification (sensitivity and specificity), and reclassification (net reclassification index). The performance of the models on internal data sets not used for model development—internal validation—and on data sets originating from an external patient population–external validation—were weighted in the ROB assignment. The ROB and applicability were assigned as *low risk, high risk,* or *unclear risk* according to PROBAST recommendations [42].

### Meta-analysis and Performance Metrics

The C statistic–area under the receiver operating curve (AUROC) is the most commonly reported estimate of discriminative performance for binary outcomes [43-46] and the pragmatic performance measure of ML and severity of illness score models previously used in the medical literature to compare models based on different computational methods [21,45-47]. It is generally interpreted as follows: an AUROC of 0.5 suggests no discrimination, 0.7 to 0.8 is considered acceptable performance, 0.8 to 0.9 is considered excellent performance, and >0.9 is considered outstanding performance [48]. We included the performance of models developed using similar algorithms in forest plots and performed heterogeneity diagnostics and investigations without calculating a pooled estimate [49]. The results were pooled only for studies that followed a consistent methodology that included the external validation or benchmarking of the models. Random-effects meta-analyses computed the pooled AUROC for the following subgroups of ML algorithms—NNs and Ensemble—and the following subgroups of scoring models—SAPS II, APACHE II and SOFA. The AUROC for each model type was weighted using the inverse of its variance. Pooled AUROC estimates for each model were meta-analyzed along with 95% CIs of the estimates and were reported in forest plots together with the associated heterogeneity statistics (*I*^2^, τ^2^, and Cochran Q). Cochran Q statistic (also known as the chi-square statistic) determines the within-study variation, τ^2^ determines the between-study variability, and *I*^2^ represents the percentage of variability from the AUROC estimate not caused by sampling error [36]. The Cochran Q *P* value is denoted as *P.* Meta-analyses were conducted in R (version 3.6.1) [37] (see Multimedia Appendix 1 for scripts).

## Results

### Selection Process

**Figure 1:**
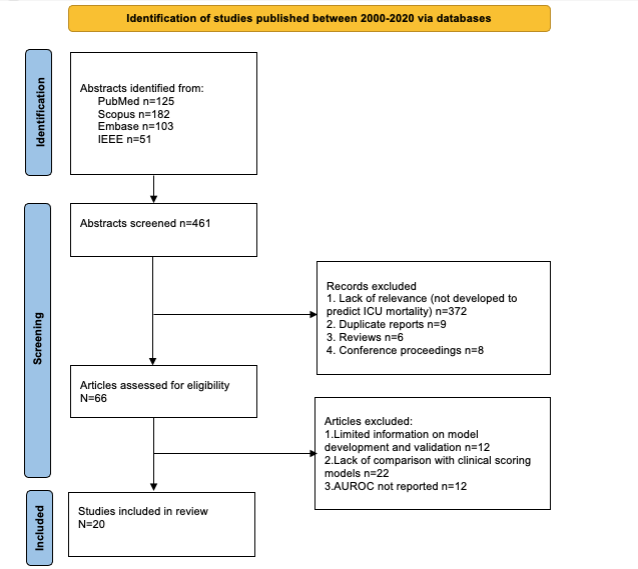
Search strategy and selection process. AUROC: area under the receiver operating curve; ICU: intensive care unit.

Of the 461 screened abstracts, we excluded 372 (80.7%) because of relevance (models not developed to predict ICU mortality), 9 (2%) duplicates, 6 (1.3%) reviews, and 8 (1.7%) conference proceedings (not intended for clinical application). We assessed the full text of 66 articles; the most common performance method reported to allow comparison between all models and a meta-analysis was the C statistic–AUROC. Of the 66 articles, we excluded 12 (18%) articles because of limited information on model development, 22 (33%) articles because of a lack of comparison with clinical scoring models, and 12 (18%) articles as the AUROC was not reported. The search strategy and selection process are illustrated in Figure 1.

### Assessment of the Prediction Model Development

The 20 studies reported 47 ML models that were developed based on 7 types of algorithms and compared them with 3 severity of illness score models. All ML models were developed through a retrospective analysis of the ICU data sets. Of the 20 studies, 10 (50%) used data from the publicly available Medical Information Mart for Intensive Care database (Beth Israel Deaconess Medical Center in the United States) at different stages of expansion. Of the 20 studies, 10 (50%) used national health care databases (Danish, Australia-New Zealand, United Kingdom, and Sweden) or ICU-linked databases (Korea, India, and the United Kingdom). One of the studies included data from >80 ICUs belonging to >40 hospitals [33], and one of the studies’ ICU-linked database collected data from 9 European countries [37]. The cohorts generating the data sets used for model development and internal testing ranged from 1571 to 217,289 patients, with a median of 15,789 patients. Of the 20 studies, 10 (50%) used data from patients admitted to general ICUs, whereas 10 (50%) studies used data from patients who were critically ill with specific pathologies: gastrointestinal bleeds [39], COVID-19 and pneumonia–associated respiratory failure [40], postcardiac arrest [28], postcardiac surgery [29,36], acute renal insufficiency [30,32], sepsis [35,41], or neurological pathology [25]. The lower age thresholds for study inclusion ranges were 12 years [25], 15 years [26,27], 16 years [33,35,38], 18 years [24,29,40], and 19 years [30]. Within the studied cohorts, mortality ranged from 0.08 to 0.5 [29,32,36].

**Figure 2:**
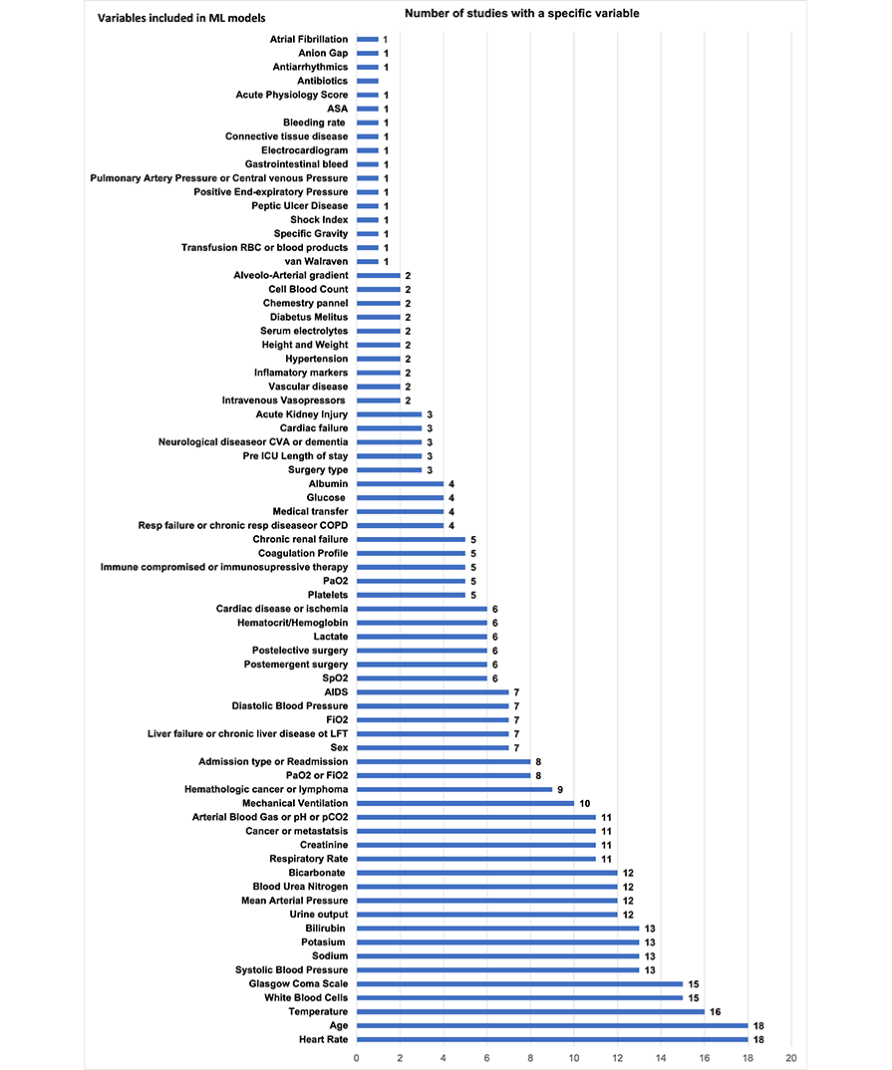
Frequency and type of ML model input variables (x-axis: number of studies using the input variables; y-axis: input variable). ASA: American Society of Anesthesiology; COPD: chronic obstructive pulmonary disease; CVA: cerebral vascular accident; FIO2: fraction of inspired oxygen; ICU: intensive care unit; LFT: liver function test; ML: machine learning; RBC: red blood cell; SpO2: oxygen saturation; PaO2: arterial oxygen pressure; PaCO2: arterial CO2 pressure.

The processes and tools used for the selection of predicting variables were described in 65% (13/20) of studies and included the least absolute shrinkage and selection operator, stochastic gradient boosting [33,35], genetic algorithms, and particle swarm optimization [33]. Approximately 15% (3/20) of studies [25,26,35] reported multiple models developed on variable predictor sets, which were subsequently tested for the best performance, validation, and calibration. The number of predictive variables used in the final models varied between 1 and 80, with a median of 21. The most common predicting variables are shown in Figure 2 and are grouped by the frequency of occurrence in the studies.

All studies developed models on 24-hour data; furthermore, ML models were developed on the first hour of ICU data [34]; the first 48-hour data [27,38,41]; 3-day data [40]; 5-day data [7]; 10-day data [26]; or on patients’ prior medical history collected from 1 month, 3 months, 6 months, 1 year, 2.5 years, 5 years, 7.5 years, 10 years, and 23 years [24]. The frequency of data collection ranged from every 30 minutes [29], 1 hour [1,25,27,37], 3 hours, 6 hours, 12 hours, 15 hours [38], and 24 hours [7,36] to every 27 hours, 51 hours, and 75 hours [40].

Researchers handled missing data and continuous and fixed variables differently. A total of 6 model developers provided no information on missing data [1,25,29,32,34,39], and 1 [27] addressed data cleaning. Researchers [24,30,33,35-37,40,41] removed the records with missing values ranging from 1 missing value per admission to 30%, 50%, and 60% of missing data. One of the studies [29] included only variables documented for at least 50% of the patients and imputed the missing values with the last measured value for the feature. Missing values (up to 60%) were forward-filled; backward-filled; or replaced with means (continuous variables) or modes (categorical variables), normal values, averages [24,28,36,38,40], predictive mean matching [7], or linear interpolation imputation method [26]. The data were normalized using the minimum-maximum normalization technique. The time prediction of hospital mortality was undefined in 45% (9/20) of studies and varied from 2 or 3 days to 28 days, 30 days [26], 90 days [24], and up to 1 year [24] in the others.

There was a wide range in the prevalence of mortality among studies (0.08-0.56), creating a class imbalance in the data sets. In studies with low investigated outcome mortality, few researchers addressed the problem of class imbalance (survivors vs nonsurvivors) through balanced training [24,37], random resampling [29], undersampling [36], or class penalty and reweighting schemes [38]. A breakdown of the model characteristics is presented in Table 3.

**Table 3:** Information on the ML^a^ prediction model development, validation, and performance, and on the severity of illness score performance.

Author	ML model type (AUROC^b^ test)	Data training/test (split %)	Features	K-fold/validation	External validation data set	ML AUROC external	Severity of illness score model type (AUROC)
Pirracchio et al, 2015 [1]	Ensemble SICULA^c^ (0.85)	24,508	17	5-fold cross-validation	200	0.94	SAPS^d^-II (0.78) APACHE^e^-II (0.83) SOF^f^ (0.71)
Nielsen et al, 2019 [24]	NN^g^ (0.792)	10,368 (80/20)	44	5-fold cross-validation	1528	0.773	SAPS-II (0.74) APACHE-II (0.72)
Nimgaonkar et al, 2004 [25]	NN (0.88)	2962 (70/30)	15	N/A^h^	N/A	N/A	APACHE-II (0.77)
Xia et al, 2019 [26]	Ensemble-LSTM^i^ (0.85) LSTM (0.83) DT^j^ (0.82)	18,415 (90/10)	50	Bootstrap and RSM^k^	N/A	N/A	SAPS-II (0.77) SOFA (0.73) APACHE-II (0.74)
Purushotham et al, 2018 [27]	NN (0.87) Ensemble (0.84)	35,627	17/22/ 136	5-fold cross-validation	External benchmark	N/A	SAPS-II (0.80) SOFA (0.73)
Nanayakkara et al, 2018 [28]	DT (0.86) SVM^l^ (0.86) NN (0.85) Ensemble (0.87) GBM^m^ (0.87)	39,560 (90/10)	29	5-fold cross-validation	N/A	N/A	APACHE-III (0.8)
Meyer et al, 2018 [29]	NN (0.95)	5898 (90/10)	52	10-fold cross-validation	5989	0.81	SAPS-II (0.71)
Meiring et al, 2018 [7]	DT (0.85) NN (0.86) SVM (0.86)	80/20	25	21,911 LOO^n^	N/A	N/A	APACHE-II (0.83)
Lin et al, 2019 [30]	DT (0.86) NN (0.83) SVM (0.86)	19,044	15	5-fold cross-validation	N/A	N/A	SAPS-II (0.79)
Krishnan et al, 2018 [31]	NN-ELM^o^ (0.99)	10,155 (75/25)	1	10-fold cross-validation	N/A	N/A	SAPS (0.80) SOFA (0.73) APS^p^-III (0.79)
Kang et al, 2020 [32]	SVM (0.77) DT (0.78) NN (0.776) k-NN^q^ (0.76)	1571 (70/30)	33	10-fold cross-validation	N/A	N/A	SOFA (0.66) APACHE-II (0.59)
Johnson et al, 2013 [33]	LR^r^ univariate (0.902) LR multivariate (0.876)	39,070 (80/20)	10	10-fold cross-validation	23,618	0.837 (univariate); 0.868 (multivariate)	APS-III (0.86)
Holmgren et al, 2019 [34]	NN (0.89)	217,289 (80/20)	8	5-fold cross-validation	N/A	N/A	SAPS-III (0.85)
Garcia-Gallo et al, 2020 [35]	SGB-LASSO^s^ (0.803)	5650 (70/30)	18 140 37	10-fold cross-validation	N/A	N/A	SOFA (0.58) SAPS (0.70)
El-Rashidy et al, 2020 [36]	Ensemble (0.93)	10,664 (75/25)	80	10-fold cross-validation	External benchmark	N/A	APACHE-II (0.73) SAPS-II (0.81) SOFA-II (0.78)
Silva et al, 2006 [37]	NN (0.85)	13,164 (66/33)	12	Hold out	N/A	N/A	SAPS- II (0.8)
Caicedo-Torres et al, 2019 [38]	NN (0.87)	22,413	22	5-fold cross-validation	N/A	N/A	SAPS-II (0.73)
Deshmukh et al, 2020 [39]	XGB^t^ (0.85)	5691 (80/20)	34	5-fold cross-validation	N/A	N/A	APACHE-IV (0.8)
Ryan et al, 2020 [40]	DT (0.86)	35,061 (80/20)	12	5-fold cross-validation	114	0.91	qSOFA^u^ (0.76)
Mayaud et al, 2013 [41]	GA^v^+LR (0.82)	2113 (70/30)	25	BBCCV^w^	N/A	N/A	APACHE-III (0.68)

^a^ML: machine learning.

^b^AUROC: area under the receiver operating curve.

^c^SICULA: Super ICU Learner Algorithm.

^d^SAPS: Simplified Acute Physiology Score.

^e^APACHE: Acute Physiology and Chronic Health Evaluation.

^f^SOFA: Sequential Organ Failure Assessment.

^g^NN: neural network.

^h^N/A: not applicable.

^i^LSTM: long short-term memory.

^j^DT: decision tree.

^k^RSM: random subspace method.

^l^SVM: support vector machine.

^m^GBM: gradient boosting machine.

^n^LOO: leave one out.

^o^ELM: extreme learning machine.

^p^APS: Acute Physiology Score.

^q^k-NN: k-nearest neighbor.

^r^LR: logistic regression.

^s^SGB-LASSO: stochastic gradient boosting least absolute shrinkage and selection operator.

^t^XGB: extreme gradient boosting.

^u^qSOFA: Quick Sequential Organ Failure Assessment.

^v^GA: genetic algorithm.

^w^BBCV: bootstrap bias–corrected cross-validation.

### Overview of ML Algorithms and Model Validation

The reviewers recorded the ML model types based on the final trained model structure rather than on the algorithm used for fitting the model (Table 3). The reviewers noted a diversity of strategies in model fitting, although the implemented models defined the operating functions and transformations. Of the 20 studies, NNs were applied in 13 (65%) [7,24-32,34,37,38], decision trees in 8 (40%) [7,26,28,30,32,35,39,40], SVM in 4 (20%) [7,28,30,32], and Ensemble of algorithms in 4 (20%) [1,27,28,36]. The types of algorithms used in the same study varied between 1 and 5. All studies provided information on data training and internal testing (see Table 2 for k-fold validation and data splitting). Of the 20 studies, 5 (25%) [1,24,29,33,40] performed validation on external data sets ranging from 114 to 23,618 patients, and 2 (10%) studies [27,36] benchmarked the ML model performance against existing ML mortality prediction models; 14 (70%) studies reported CIs for the measure of discrimination AUROC, 9 (45%) studies reported on calibration (Hosmer-Lemeshow, calibration curve, or Brier score), and 12 (60%) studies reported on classification measures (Table 4). Approximately 10% (2/20) of studies were available for use in clinical practice [1,33]; the models’ decisions were explained with local interpretable model-agnostic explanations [28] or the Shapley additive explanations method (SHAP) [39]. The AUROC of the ML models ranged from 0.728 to 0.99 for predicting mortality.

**Table 4:** Reported performance measures of the ML^a^ models.

Author and ML model		Classification measurements						Calibration measurements				Other
		Specificity	PPV^b^/precision	Recall/sensitivity	F_1_ score	Accuracy	HL^c^ score		Brier score	Calibration curve		
**Pirrachio et al** **[1]**												
	Ensemble SL^d^-1	N/A^e^	N/A	N/A	N/A	N/A	N/A		0.079	*U*^f^=0.0007 (calibration plot)	DS^g^=0.21	
	Ensemble SL-2	N/A	N/A	N/A	N/A	N/A	N/A		0.079	*U*=0.006 (calibration plot)	DS=0.26	
**Nielsen et al** **[24]**												
	NN^h^	N/A	0.388	N/A	N/A	N/A	N/A		N/A	N/A	Mathews correlation coefficient	
**Purushotham et al** **[27]**												
	NN	N/A	N/A	N/A	N/A	N/A	N/A		N/A	N/A	0.491 (AUPRC^i^)	
	Ensemble	N/A	N/A	N/A	N/A	N/A	N/A		N/A	N/A	0.435 (AUPRC)	
**Nimgaonkar et** **al [25]**												
	NN-15 features	N/A	N/A	N/A	N/A	N/A	27.7		N/A	Calibration plot	N/A	
	NN-22 features	N/A	N/A	N/A	N/A	N/A	22.4		N/A	Calibration plot	N/A	
**Xia et al** **[26]**												
	Ensemble-LSTM^j^	0.7503	0.294	0.7758	0.4262	0.7533	N/A		N/A	N/A	N/A	
	LSTM	0.7746	0.305	0.7384	0.4317	0.7703	N/A		N/A	N/A	N/A	
	RF^k^	0.7807	0.306	0.71197	0.4290	0.7734	N/A		N/A	N/A	N/A	
**Nanayakkara et al** **[28]**												
	RF	0.79	0.75	0.76	N/A	0.78	N/A		0.156	Calibration plot	0.47 (log loss)	
	SVC^l^	0.81	0.77	0.75	N/A	0.78	N/A		0.153	Calibration plot	0.47 (log loss)	
	GBM^m^	0.78	0.75	0.8	N/A	0.79	N/A		0.147	Calibration plot	0.45 (log loss)	
	NN	0.72	0.71	0.82	N/A	0.77	N/A		0.158	Calibration plot	0.48 (log loss)	
	Ensemble	0.81	0.77	0.77	N/A	0.79	N/A		0.148	Calibration plot	0.45 (log loss)	
**Meyer et al** **[29]**												
	RNN^n^	0.91	0.9	0.85	0.88	0.88	N/A		N/A	N/A	N/A	
**Meiring et al** **[7]**												
	DT^o^, NN, SVM^p^	N/A	N/A	N/A	N/A	N/A	N/A		N/A	N/A	N/A	
**Lin et al** **[30]**												
	RF	N/A	N/A	N/A	0.459	0.728	N/A		0.085	Calibration plot	N/A	
	NN	N/A	N/A	N/A	0.406	0.666	N/A		0.091	Calibration plot	N/A	
	SVM	N/A	N/A	N/A	0.460	0.729	N/A		0.086	Calibration plot	N/A	
**Krishnan et al** **[31]**												
	ANN-ELM^q^	N/A	N/A	0.98	0.98	0.98	N/A		N/A	N/A	Mathews correlation coefficient	
**Kang et al** **[32]**												
	k-NN^r^	N/A	N/A	N/A	0.745	0.673	N/A		N/A	Calibration plot	N/A	
	SVM	N/A	N/A	N/A	0.752	0.696	N/A		N/A	Calibration plot	N/A	
	RF	N/A	N/A	N/A	0.762	0.69	N/A		N/A	Calibration plot	N/A	
	XGB^s^	N/A	N/A	N/A	0.763	0.711	N/A		N/A	Calibration plot	N/A	
	NN	N/A	N/A	N/A	0.749		N/A		N/A	Calibration plot	N/A	
**Johnson et al** **[33]**												
	LR^t^ univariate	N/A	N/A	N/A	N/A	N/A	22		0.051	N/A	N/A	
	LR multivariate	N/A	N/A	N/A	N/A	N/A	19.6		0.048	N/A	N/A	
**Holmgren et al** **[34]**												
	NN	N/A	N/A	N/A	N/A	N/A	N/A		0.106	Calibration plot	N/A	
**Garcia-Gallo et al** **[35]**												
	SGB^u^	N/A	N/A	N/A	N/A	0.725	0.0916		N/A	Calibration plot	N/A	
	SGB-LASSO^v^	N/A	N/A	N/A	N/A	0.712	0.0916		N/A	Calibration plot	N/A	
**El-Rashidy et al** **[36]**												
	Ensemble	0.94	N/A	0.911	0.937	0.944	N/A		N/A	N/A	N/A	
**Silva et al** **[37]**												
	NN	0.79	N/A	0.78	N/A	0.7921	N/A		N/A	N/A	N/A	
**Caicedo-Torres et al** **[38]**												
	NN	0.827	N/A	0.75	N/A	N/A	N/A		N/A	N/A	N/A	
**Deshmukh et al** **[39]**												
	XGB	0.27	N/A	1	N/A	N/A	N/A		N/A	N/A	N/A	
**Ryan et al** **[40]**												
	XGB	0.75	N/A	0.801	0.378	0.75	N/A		N/A	N/A	N/A	
**Mayaud et al** **[41]**												
	GA^w^+LR	N/A	N/A	N/A	N/A	N/A	10.43		N/A	Calibration plot	N/A	

^a^ML: machine learning.

^b^PPV: positive predictive value.

^c^HL: Hosmer-Lemeshow.

^d^SL: super learner.

^e^N/A: not available.

^f^*U* statistics.

^g^DS: discrimination slope.

^h^NN: neural network.

^i^AUPRC: area under the precison-recall curve.

^j^LSTM: long short-term memory.

^k^RF: random forest.

^l^SVC: support vector classifier.

^m^GBM: gradient boosting machine.

^n^RNN: recurrent neural network.

^o^DT: decision tree.

^p^SVM: support vector machine.

^q^ANN-ELM: artificial neural network extreme learning machine.

^r^k-NN: k-nearest neighbor.

^s^XGB: extreme gradient boosting.

^t^LR: logistic regression.

^u^SGB: stochastic gradient boosting.

^v^LASSO: least absolute shrinkage and selection operator.

^w^GA: genetic algorithm.

The performance of the ML models was compared with that of the following severity of illness scoring models: APACHE-II (6/20, 30%), APACHE-III (2/20, 10%), APACHE-IV (1/20, 5%), SAPS-II (11/20, 55%), SAPS-III (1/20, 5%), SOFA (9/20, 45%), and Acute Physiology Score-3 (2/20, 10%; Table 3). The severity of illness scores’ discrimination reported as AUROC was associated with a CI in 65% (13/20) of studies. Calibration of the severity of illness score models was reported in 30% (6/20) of studies. Approximately 60% (12/20) of studies reported binary classification results. The severity of illness scores used for comparison and associated AUROCs were 0.70 to 0.803 for SAPS, 0.588 to 0.782 for SOFA, and 0.593 to 0.86 for APACHE (Table 3).

### Analysis of ROB and Applicability

The results of the analysis of the ROB in the selection of the study population, predictors, outcome definition, and performance reporting are presented in Table 5. The results of the assessment of the developed ML models’ applicability regarding the study participants and setting, the predictors used in the ML models’ development and their timing, the outcome definition and prediction by the models, and the analysis that reports the models’ performance are also presented in Table 5. Of the 47 models, 4 (9%) models [1,17,23,29] were identified as having a low risk, and 3 (6%) models were rated as having an uncertain ROB and applicability model development [24,29,40]. The main reason for the high ROB in the overall judgment of the study was the lack of external validation, which was identified in 28% (13/47) of the models.

**Table 5:** Assessment for ROB^a^ and applicability for prognostic models with the Prediction model ROB Assessment Tool checklist.

Authors	ROB and applicability										
	Participants		Predictors		Outcome			Analysis		Overall judgment	
	ROB	Applicability	ROB	Applicability	ROB	Applicability	ROB		ROB		Applicability
Pirracchio et al [1]	Low^b^	Low	Low	Low	Low	Low	Low		Low		Low
Nielsen et al [24]	Low	Low	Unclear^c^	Low	Unclear	Low	Low		Unclear		Low
Nimgaonkar et al [25]	Low	Unclear	Low	Low	Low	Low	High^d^		High		Unclear
Xia et al [26]	Low	Low	Low	Low	Unclear	Low	High		High		Low
Purushotham et al [27]	Low	Low	Low	Low	Low	Low	Low		Low		Low
Nanayakkara et al [28]	Low	Unclear	Low	Low	Low	Low	High		High		Unclear
Meyer et al [29]	Low	Unclear	Low	Low	Low	Low	Low		Low		Unclear
Meiring et al [7]	Low	Low	Low	Low	Low	Low	High		High		Low
Lin et al [30]	Low	Unclear	Low	Low	Low	Low	High		High		Unclear
Krishnan et al [31]	Low	Low	Low	Low	Low	Low	High		High		Low
Kang et al [32]	Low	Unclear	Low	Low	Low	Low	High		High		Unclear
Johnson et al [33]	Low	Low	Low	Low	Low	Low	Low		Low		Low
Holmgren et al [34]	Low	Low	Low	Low	Unclear	Low	High		High		Low
Garcia-Gallo et al [35]	Low	Unclear	Low	Low	Low	Low	High		High		Unclear
El-Rashidy et al [36]	Low	Low	Low	Low	Low	Low	Low		Low		Low
Silva et al [37]	Low	Low	Low	Low	Low	Low	High		High		Low
Caicedo-Torres et al [38]	Low	Low	Low	Low	Low	Low	High		High		Low
Deshmukh et al [39]	Low	Unclear	Low	Low	Low	Low	High		High		Unclear
Ryan et al [40]	Low	Low	Unclear	Low	Low	Low	Low		Unclear		Low
Mayaud et al [41]	Low	Unclear	Low	Unclear	Low	Low	High		High		Unclear

^a^ROB: risk of bias.

^b^Low risk: no relevant shortcomings in ROB assessment.

^c^Unclear risk: unclear ROB in at least one domain and all other domains at low ROB.

^d^High risk: relevant shortcomings in the ROB assessment, at least one domain with high ROB, or model developed without external validation.

### Meta-analysis

**Figure 3:**
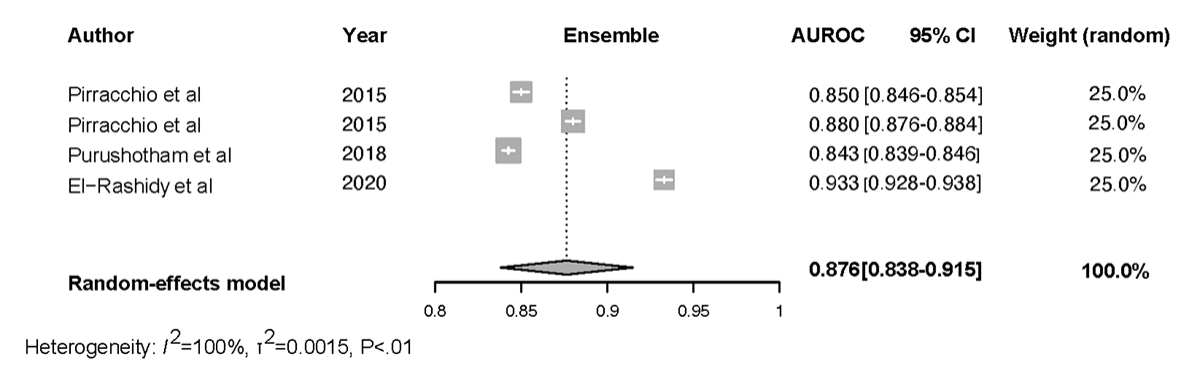
Meta-analysis results: pooled AUROC for externally validated Ensemble models. Gray boxes represent the fixed weight estimates of the AUROC value from each study. Larger gray boxes represent larger fixed weight estimates of the AUROC values. The horizontal line through each gray box illustrates the 95% CI of the AUROC value from that study. Black horizontal lines through a gray box indicate that the CI limits exceed the length of the gray box. White horizontal lines represent CI limits that are within the length of the gray box. The vertical dashed lines in the forest plot are the estimated random pooled effect of the AUROC value from the random-effects meta-analysis. The gray diamonds illustrate the 95% CI for the random pooled effects. Tests of heterogeneity included *I*^2^, τ^2^, and Cochran Q *P* value (denoted as P). AUROC: area under the receiver operating curve;.

**Figure 4:**
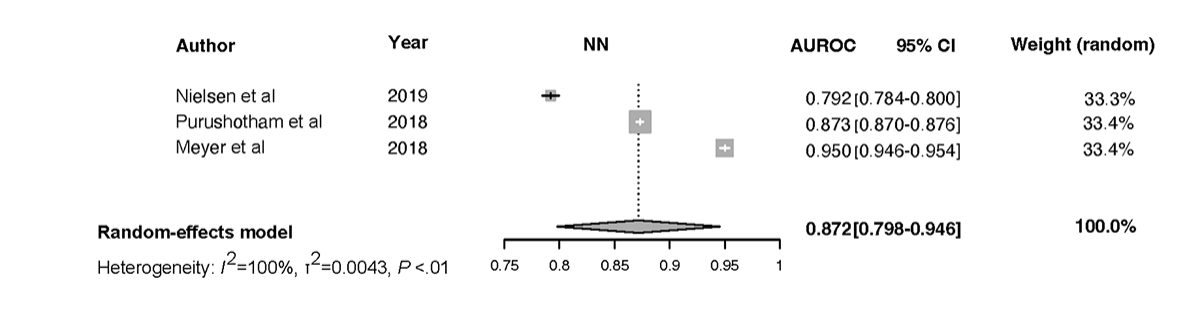
Meta-analysis results: pooled AUROC for externally validated NN models. Gray boxes represent the fixed weight estimates of the AUROC value from each study. Larger gray boxes represent larger fixed weight estimates of the AUROC values. The horizontal line through each gray box illustrates the 95% CI of the AUROC value from that study. Black horizontal lines through a gray box indicate that the CI limits exceed the length of the gray box. White horizontal lines represent CI limits that are within the length of the gray box. The vertical dashed lines in the forest plot are the estimated random pooled effect of the AUROC value from the random-effects meta-analysis. The gray diamonds illustrate the 95% CI for the random pooled effects. Tests of heterogeneity included *I*^2^, τ^2^, and Cochran Q *P* value (denoted as P). AUROC: area under the receiver operating curve; NN: neural network.

**Figure 5:**
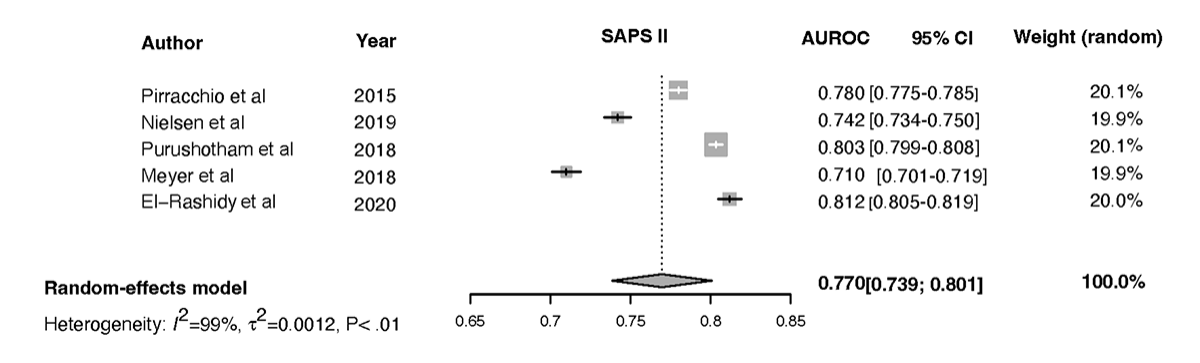
Meta-analysis results: pooled AUROC for SAPS-II. Gray boxes represent the fixed weight estimates of the AUROC value from each study. Larger gray boxes represent larger fixed weight estimates of the AUROC values. The horizontal line through each gray box illustrates the 95% CI of the AUROC value from that study. Black horizontal lines through a gray box indicate that the CI limits exceed the length of the gray box. White horizontal lines represent CI limits that are within the length of the gray box. The vertical dashed lines in the forest plot are the estimated random pooled effect of the AUROC value from the random-effects meta-analysis. The gray diamonds illustrate the 95% CI for the random pooled effects. Tests of heterogeneity included *I*^2^, τ^2^, and Cochran Q *P* value (denoted as P). AUROC: area under the receiver operating curve; SAPS-II: Simplified Acute Physiology Score II.

**Figure 6:**
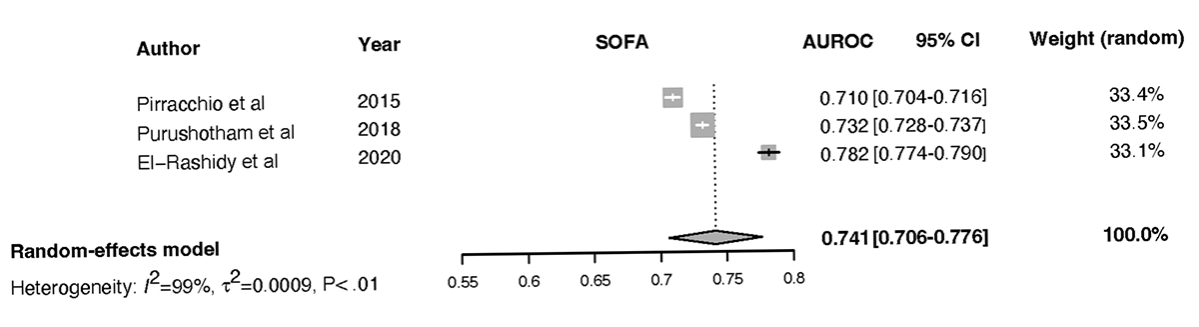
Meta-analysis results: pooled AUROC for SOFA. Gray boxes represent the fixed weight estimates of the AUROC value from each study. Larger gray boxes represent larger fixed weight estimates of the AUROC values. The horizontal line through each gray box illustrates the 95% CI of the AUROC value from that study. Black horizontal lines through a gray box indicate that the CI limits exceed the length of the gray box. White horizontal lines represent CI limits that are within the length of the gray box. The vertical dashed lines in the forest plot are the estimated random pooled effect of the AUROC value from the random-effects meta-analysis. The gray diamonds illustrate the 95% CI for the random pooled effects. Tests of heterogeneity included *I*^2^, τ^2^, and Cochran Q *P* value (denoted as P). AUROC: area under the receiver operating curve; SOFA: Sequential Organ Failure Assessment.

**Figure 7:**
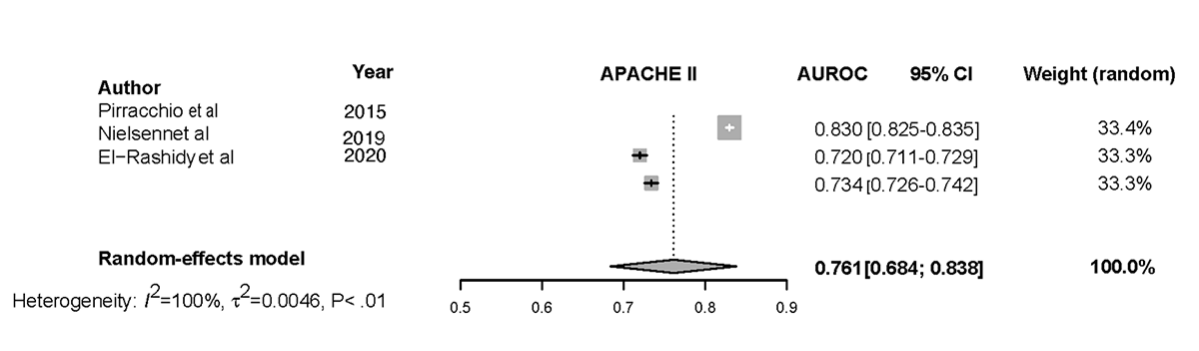
Meta-analysis results: pooled AUROC for APACHE-II. Gray boxes represent the fixed weight estimates of the AUROC value from each study. Larger gray boxes represent larger fixed weight estimates of the AUROC values. The horizontal line through each gray box illustrates the 95% CI of the AUROC value from that study. Black horizontal lines through a gray box indicate that the CI limits exceed the length of the gray box. White horizontal lines represent CI limits that are within the length of the gray box. The vertical dashed lines in the forest plot are the estimated random pooled effect of the AUROC value from the random-effects meta-analysis. The gray diamonds illustrate the 95% CI for the random pooled effects. Tests of heterogeneity included *I*^2^, τ^2^, and Cochran Q *P* value (denoted as P). APACHE-II: Acute Physiology and Chronic Health Evaluation-II; AUROC: area under the receiver operating curve;.

Forest plots for the NN, Ensemble, SOFA, SAPS II, and APACHE-II models and the associated heterogeneity tests are shown in Figures 3-7. The forest plots and tests of heterogeneity for SVM, NN, DT, and Ensemble models that were not externally validated can be seen in Multimedia Appendix 2. The AUROC for each model type was weighted using the inverse of its variance. Most of the 95% CIs of AUROC estimates from various studies did not overlap within the forest plot; considerable variation among AUROC estimates for both ML and severity of illness score model types was noted. Regrading tests of heterogeneity, *I*^2^ varied between 99% and 100%, τ^2^ ranged from 0.0003 to 0.0034, and *P* was consistently <.01. In Figures 3-7 and Multimedia Appendix 2, the gray boxes represent the weight estimates of the AUROC value from each study. The horizontal line through each gray box illustrates the 95% CI of the AUROC value from that study. Black horizontal lines through a gray box indicate that the CI limits exceeded the length of the gray box. White horizontal lines represent the CI limits that are within the length of the gray box. *I^2^*, τ^2^, and Cochran Q *P* value (denoted as *P*) are heterogeneity tests.

Random-effects meta-analysis results of the computed pooled AUROC of the ML subgroup models that were externally validated or benchmarked NNs and Ensemble are shown in Figure 3 and Figure 4.

The results of heterogeneity for the NN models were as follows: τ^2^= 0.0043 (95% CI 0.0014-0.2100), *I*^2^=99.9% (95% CI 99.8%-99.9%), *P*<.01. The results of heterogeneity for the Ensemble models were as follows:

τ^2^=0.0015 (95% CI 0.0005-0.0223), *I*^2^=99.7% (95% CI 99.6%-99.8%), *P*<.01. The results were synthesized, and the models are presented in Figure 3 and Figure 4. The results of heterogeneity for the APACHE-2 models were as follows: τ^2^=0.0046 (95% CI 0.0011-0.1681), *I*^2^=99.7% (95% CI 99.6%-99.8%), *P*<.01. The results of heterogeneity for the SAPS-II models were as follows: τ^2^=0.0012 (95% CI 0.0005-0.0133), *I*^2^=99.2% (95% CI 98.9%-99.4%), *P*<.01. The results of heterogeneity for the SOFA models were as follows: τ^2^=0.0009 (95% CI 0.0003-0.0461), *I*^2^=99.1% (95% CI 98.5%-99.4%), *P*<.01 (Figures 5-7).

## Discussion

### Principal Findings

This is the first study to critically appraise the literature comparing the ML and severity of illness score models to predict ICU mortality. In the reviewed articles, the AUROC of the ML models demonstrated very good discrimination. The range of the ML model AUROC was superior to that of the severity of illness score AUROC. The meta-analysis demonstrated a high degree of heterogeneity and variability within and among studies; therefore, the AUROC performances of the ML and severity of illness score models cannot be pooled, and the results cannot be generalized. Every *I*^2^ value is >97.7%; most of the 95% CIs of AUROC estimates from various studies did not overlap within the forest plot, suggesting considerable variation among AUROC estimates for model types. The CI for AUROC and the statistical significance of the difference in model performance were inconsistently reported within studies. The high heterogeneity came from the diverse study population and practice location, age of inclusion, primary pathology, medical management leading to the ICU admission, and time prediction window. The heterogenous data management (granularity, frequency of data input, data management, number of predicting variables, prediction timeframe, time series analysis, and training set imbalance) affected model development. It may have resulted in bias, primarily in studies where it has not been addressed (Table 2). Generally, authors reported the ML algorithms with predictive power superior to the clinical scoring system (Table 3); the number of ML models with inferior performance not reported is unknown, which raises the concern of reporting bias. The classification measures of performance were inconsistently reported and required a predefined probability threshold; therefore, models showed different sensitivity and specificity based on the chosen threshold. The variations in the prevalence of the studied outcome secondary to imbalanced data sets make the interpretation of the accuracy difficult. The models’ calibration cannot be interpreted because of limited reporting. The external validation process that is necessary to establish generalization was lacking in 65% (13/20) of studies (Table 2). The limited and variable performance metrics reported precludes a comprehensive model performance comparison among studies. The decision curve analysis and model interpretability (explainability) that are necessary to promote transparency and understanding of the model’s predictive reasoning was addressed in 25% (5/20) of studies. Results of the clinical performance of ML mortality prediction models as alternatives to the severity of illness score are scarce.

The reviewed studies inconsistently and incompletely captured the descriptive characteristics and other method parameters for ML-based predictive model development. Therefore, we cannot fully assess the superiority or inferiority of ML-based ICU mortality prediction compared with traditional models; however, we recognize the advantage that flexibility in model design offers in the ICU setting.

### Study Limitations

This review included studies that were retrospective analyses of data sets with known outcome distributions and incorporated the results of interventions. It is unclear which models were developed exclusively for research purposes; hence, they were not validated. We evaluated studies that compared ML-based mortality prediction models with the severity of illness score–based models, although these models relied on different development statistical methods, variable collection times, and outcome measurement methodologies (SOFA).

The comparison between the artificial intelligence (AI) and severity of illness score models relies only on AUROC values as measures of calibration, discrimination, and classification are not uniformly reported. The random-effects meta-analysis was limited to externally validated models. Owing to the level of heterogeneity, the performance results for most AI and severity of illness score models could not be pooled. The authors recognize that 25% (5/20) of the articles were published between 2004 and 2015 before the TRIPOD (Transparent Reporting of a multivariable prediction model for Individual Prognosis or Diagnosis) recommendations for model development and reporting [18]; thus, they were not aligned with the guidelines.

The reviewers assessed the models’ ROB and applicability and were aware of the risk of reporting and publication bias favoring the ML models. However, the high heterogeneity among studies prevents an unambiguous interpretation of the funnel plot.

### Conclusions and Recommendations

The results of our analysis show that the reporting methodology is incomplete, nonadherent to the current recommendations, and consistent with previous observations [16,50]. The lack of consistent reporting of the measures of the reliability calibration (Brier score and calibration curve of reliability deviation), discrimination, and classification of the probabilistic estimates on external data makes the comparative effectiveness of risk prediction models challenging and has been noted by other authors [43].

Predictive models of mortality can substantially increase patient safety, and by incorporating subtle changes in organ functions that affect outcomes, these models support the early recognition and diagnosis of patients who are deteriorating, thus providing clinicians with additional time to intervene. The heterogeneity of the classification models that was revealed in detail in this review underlines the importance of recognizing the models’ ability for temporal and geographical generalization or proper adaptation to previously unseen data [51]. These concepts apply to both models; similar to the ML models, severity of illness score requires periodical updates and customizations to reflect changes in medical care and regional case pathology over time [6].

Our findings lead to the following recommendations for model developers:

1. State whether the developed ML models are intended for clinical practice
2. If models are intended for clinical applications, provide full transparency of the clinical setting from which the data are acquired and all the model development steps; validate the models externally to ensure generalizability
3. If intended for clinical practice, report models’ performance metrics, which include measures of discrimination, calibration, and classification, and attach explainer models to facilitate interpretability

Before using ML and/or severity of illness score models as decision support systems to guide clinical practice, we make the following recommendations for clinicians:

1. Be cognizant of the similarities or discrepancies between the cohort used for model development and the local practice population, the practice setting, the model’s ability to function prospectively, and the models’ lead times
2. Acquire knowledge of the model’s performance during testing in the local practice
3. Ensure that the model is periodically updated to changes in patient characteristics and/or clinical variables and adjusted to new clinical practices and therapeutics
4. Confirm that the models’ data are monitored and validated and that the model’s performance is periodically updated
5. When both the severity of illness score and ML models are available, determine one model’s superiority and clinical reliability versus the other through randomized controlled trials
6. When ML models guide clinical practice, ensure that the model makes the correct recommendation for the
  right reasons
  and consult the explainer model
7. Identify clinical performance metrics that evaluate the impact of the AI tool on the quality of care, efficiency, productivity, and patient outcomes and account for variability in practice

AI developers must search for and clinicians must be cognizant of the unintended consequences of AI tools; both must understand human–AI tool interactions. Healthcare organization administrators must be aware of the safety, privacy, causality, and ethical challenges when adopting AI tools and recognize the Food and Drug Administration guiding principles for AI/ML development [52].

## Appendix

Multimedia Appendix 1 R scripts for meta-analysis.

Multimedia Appendix 2 Forest plots for neural network, decision tree, support vector machine, and Ensemble-based models.

## Abbreviations

AI: artificial intelligence
APACHE: Acute Physiology and Chronic Health Evaluation
AUROC: area under the receiver operating curve
CHARMS: Critical Appraisal and Data Extraction for Systematic Reviews of Prediction Modeling Studies
ICU: intensive care unit
ML: machine learning
NN: neural network
PRISMA: Preferred Reporting Items for Systematic Reviews and Meta-Analyses
PROBAST: Prediction model Risk of Bias Assessment Tool
ROB: risk of bias
SAPS: Simplified Acute Physiology Score
SOFA: Sequential Organ Failure Assessment
SVM: support vector machine
TRIPOD: Transparent Reporting of a multivariable prediction model for Individual Prognosis or Diagnosis
