# Supplementary material for: Comparison of Severity of Illness Scores and Artificial Intelligence Models That Are Predictive of Intensive Care Unit Mortality: Meta-analysis and Review of the Literature
Source: JMIR Med Inform. 2022 May 31;10(5):e35293. doi: 10.2196/35293 (PMC9198821; doi:10.2196/35293)
Supplement: Multimedia Appendix 1 [file medinform_v10i5e35293_app1.pdf]

Multimedia Appendix 1

```
#The R code below includes that meta-analysis for the following:
#a)AI categories/types Meta-Analysis
#b)CM Model Meta-Analysis
#c) Meta-Analysis: Difference between maximum AI AUROC - CM AUROC by type
(SAPS, SOFA, and APACHE/APS)
###a)AI categories/types Meta-Analysis: AI model meta analysis by AI
categories/type (i.e. decision tree, ensemble, etc.)
AI_model <-
read_excel("R:\\PrevMed\\Projects\\BCC\\Projects\\BarboiC_1416\\Analysis\\
Data\\AI model for analysis.xlsx")

View(AI_model)
table(AI_model$AI_Category)
Decisiontree_data<- AI_model[ which(AI_model$AI_Category =='Decision Tree
(DT)'|AI_model$AI_Category=='Decision Tree(DT)'| AI_model$AI_Category
=='DT'|AI_model$AI_Category =='DT 48'|AI_model$AI_Category
=='DT48'|AI_model$AI_Category =='DT72'),]
AI_Decisiontree<- metaprop( Numerator_decimal,SampleSize ,studlab=Author,
                            sm="PRAW",
dataDecisiontree_data,method="Inverse", method.tau="DL", method.ci =
"NAsm")
AI_Decisiontree
AI_Decisiontree$pval.Q
forest(AI_Decisiontree,  xlim = c(.72,.9),comb.fixed=TRUE,
comb.random=TRUE,
       comb.r=T, comb.f=T,
       fontsize=7.8, digits=3,boxsize=0.8, digits.pval =
max(gs("digits.pval") - 2, 2),
       leftcols=c("studlab", "Year", "AI model","SampleSize"),
       leftlabs=c("Author", "Year", "AI Model Type", "Training and
Validation Set"),
       rightlabs=c( "AUROC","95% CI", "Weight (fixed)", "Weight
(random)"))
#DT no 48 or 72
DecisiontreeOnly_data<- AI_model[ which(AI_model$AI_Category =='Decision
Tree (DT)'|AI_model$AI_Category=='Decision Tree(DT)'| AI_model$AI_Category
=='DT'),]

AI_DecisiontreeOnly<- metaprop( Numerator_whole,SampleSize
,studlab=Author,
                                sm="PRAW", data =
DecisiontreeOnly_data,method="Inverse", method.tau="DL", method.ci =
"NAsm")
forest(AI_DecisiontreeOnly, xlim = c(.72,.9),comb.fixed=TRUE,
comb.random=FALSE,
       comb.r=T, comb.f=F,
       fontsize=10, digits=3,boxsize=0.8,
       rightlabs=c("AUC", "95% CI","weight"),  leftcols="studlab")
##forest(AI_DecisiontreeOnly, xlim = c(.72,.9),comb.fixed=TRUE,
comb.random=TRUE,
       comb.r=T, comb.f=T,
       fontsize=8, digits=3,boxsize=0.8,digits.pval =
max(gs("digits.pval") - 2, 2),
```

```
  leftcols=c("studlab", "Year", "AI model","SampleSize"),
       leftlabs=c("Author", "Year", "AI Model Type", "Training and
Validation Set"),
       rightlabs=c( "AUROC","95% CI", "Weight (fixed)", "Weight
(random)"))
#Neural networks meta analysis
AI_model <-
read_excel("R:\\PrevMed\\Projects\\BCC\\Projects\\BarboiC_1416\\Analysis\\
Data\\AI model for analysis.xlsx")

table(AI_model$AI_Category)
NN_data<- AI_model[ which(AI_model$AI_Category =='Neural Network
(NN)'|AI_model$AI_Category=='Neural Network(NN)'| AI_model$AI_Category
=='NN48'|AI_model$AI_Category =='Recurrent Neural Network(NN)'),]

AI_NN<- metaprop( Numerator_decimal,SampleSize ,studlab=Author,
                  sm="PRAW", data = NN_data,method="Inverse",
method.tau="DL", method.ci = "NAsm")

AI_NN
AI_NN$pval.Q

forest(AI_NN,  xlim = c(.72,1),comb.fixed=TRUE, comb.random=TRUE,
       comb.r=T, comb.f=T,
       fontsize=7.8, digits=3,boxsize=0.8, digits.pval =
max(gs("digits.pval") - 2, 2),
       leftcols=c("studlab", "Year", "AI model","SampleSize"),
       leftlabs=c("Author", "Year", "AI Model Type", "Training and
Validation Set"),
       rightlabs=c( "AUROC","95% CI", "Weight (fixed)", "Weight
(random)"))
AI_model <-
read_excel("R:\\PrevMed\\Projects\\BCC\\Projects\\BarboiC_1416\\Analysis\\
Data\\AI model for analysis.xlsx")

table(AI_model$AI_Category)
Esemble_data<- AI_model[ which(AI_model$AI_Category
=='Ensemble(E)'|AI_model$AI_Category=='Ensemble (E)'),]

AI_Esemble<- metaprop( Numerator_decimal,SampleSize ,studlab=Author,
                       sm="PRAW", data = Esemble_data,method="Inverse",
method.tau="DL", method.ci = "NAsm")

AI_Esemble
AI_Esemble$pval.Q

forest(AI_Esemble,  xlim = c(.8,1),comb.fixed=TRUE, comb.random=TRUE,
       comb.r=T, comb.f=T,
       fontsize=7.8, digits=3,boxsize=0.8, digits.pval =
max(gs("digits.pval") - 2, 2),
       leftcols=c("studlab", "Year", "AI model","SampleSize"),
       leftlabs=c("Author", "Year", "AI Model Type", "Training and
Validation Set"),
```

```
      rightlabs=c( "AUROC","95% CI", "Weight (fixed)", "Weight
(random)"))#SVM meta analysis--NEED TO DO THIS
AI_model <-
read_excel("R:\\PrevMed\\Projects\\BCC\\Projects\\BarboiC_1416\\Analysis\\
Data\\AI model for analysis.xlsx")
table(AI_model$AI_Category)
SVM_data<- AI_model[ which(AI_model$AI_Category =='Support Vector
Machine(SVM)'|AI_model$AI_Category=='SVM48'),]

AI_SVM<- metaprop( Numerator_decimal,SampleSize ,studlab=Author,
                   sm="PRAW", data = SVM_data,method="Inverse",
method.tau="DL", method.ci = "NAsm")AI_SVM
AI_SVM$pval.Q
forest(AI_SVM,  xlim = c(.70,1),comb.fixed=TRUE, comb.random=TRUE,
       comb.r=T, comb.f=T,
       fontsize=7.8, digits=3,boxsize=0.8, digits.pval =
max(gs("digits.pval") - 2, 2),
       leftcols=c("studlab", "Year", "AI model","SampleSize"),
       leftlabs=c("Author", "Year", "AI Model Type", "Training and
Validation Set"),
       rightlabs=c( "AUROC","95% CI", "Weight (fixed)", "Weight
(random)"))
#Recurrent Neural Network meta analysis
AI_model <-
read_excel("R:\\PrevMed\\Projects\\BCC\\Projects\\BarboiC_1416\\Analysis\\
Data\\AI model for analysis.xlsx")
table(AI_model$AI_Category)
RecurrNN_data<- AI_model[ which(AI_model$AI_Category =='Recurrent Neural
Network(NN)'),]

AI_RecurrNN<- metaprop( Numerator_decimal,SampleSize ,studlab=Author,
                        sm="PRAW", data = RecurrNN_data,method="Inverse",
method.tau="DL", method.ci = "NAsm")

AI_RecurrNN
forest(AI_RecurrNN,  xlim = c(.80,1),comb.fixed=TRUE, comb.random=TRUE,
       comb.r=T, comb.f=T,
       fontsize=7.8, digits=3,boxsize=0.8, digits.pval =
max(gs("digits.pval") - 2, 2),
       leftcols=c("studlab", "Year", "AI model","SampleSize"),
       leftlabs=c("Author", "Year", "AI Model Type", "Training and
Validation Set"),
       rightlabs=c( "AUROC","95% CI", "Weight (fixed)", "Weight
(random)"))
#GA+LR
AI_model <-
read_excel("R:\\PrevMed\\Projects\\BCC\\Projects\\BarboiC_1416\\Analysis\\
Data\\AI model for analysis.xlsx")

table(AI_model$AI_Category)
GALR_data<- AI_model[ which(AI_model$AI_Category
=='GA+LR'|AI_model$AI_Category =='GA+ LR'),]
AI_GALR<- metaprop( Numerator_decimal,SampleSize ,studlab=Author,
```

```
                    sm="PRAW", data = GALR_data,method="Inverse",
method.tau="DL", method.ci = "NAsm")
AI_GALR
forest(AI_GALR,  xlim = c(.75,.95),comb.fixed=TRUE, comb.random=TRUE,
       comb.r=T, comb.f=T,
       fontsize=7.8, digits=3,boxsize=0.8, digits.pval =
max(gs("digits.pval") - 2, 2),
       leftcols=c("studlab", "Year", "AI model","SampleSize"),
       leftlabs=c("Author", "Year", "AI Model Type", "Training and
Validation Set"),
       rightlabs=c( "AUROC","95% CI", "Weight (fixed)", "Weight
(random)")) )CM Model Meta-Analysis
CM_model <-
read_excel("R:\\PrevMed\\Projects\\BCC\\Projects\\BarboiC_1416\\Analysis\\
Data\\CM model for analysis.xlsx")
View(CM_model)
table(CM_model$`Comparison Model (CM)`)
CM_model <-
read_excel("R:\\PrevMed\\Projects\\BCC\\Projects\\BarboiC_1416\\Analysis\\
Data\\CM model for analysis.xlsx")

Apache2_data <- CM_model[ which(CM_model$`Comparison Model (CM)` =='APACHE
II'|CM_model$`Comparison Model (CM)`=='APACHEII'),]

CM_Apache2 <- metaprop( Numerator_decimal,SampleSize ,studlab=Author,
                        sm="PRAW", data = Apache2_data,method="Inverse",
method.tau="DL", method.ci = "NAsm")

CM_Apache2
CM_model$`Comparison Model (CM)`
forest(CM_Apache2,  xlim = c(.5,.9),comb.fixed=TRUE, comb.random=TRUE,
       comb.r=T, comb.f=T,
       fontsize=7.8, digits=3,boxsize=0.8, digits.pval =
max(gs("digits.pval") - 2, 2),
       leftcols=c("studlab", "Year", "Comparison Model
(CM)","SampleSize"),
       leftlabs=c("Author", "Year", "CM Model Type", "Training and
Validation Set"),
       rightlabs=c( "AUROC","95% CI", "Weight (fixed)", "Weight
(random)"))
CM_model <-
read_excel("R:\\PrevMed\\Projects\\BCC\\Projects\\BarboiC_1416\\Analysis\\
Data\\CM model for analysis.xlsx")
Apache3_data <- CM_model[ which(CM_model$`Comparison Model (CM)` =='APACHE
III'),]
CM_Apache3<- metaprop( Numerator_decimal,SampleSize ,studlab=Author,
                       sm="PRAW", data = Apache3_data,method="Inverse",
method.tau="DL", method.ci = "NAsm")

CM_Apache3
CM_model$`Comparison Model (CM)`
forest(CM_Apache3,  xlim = c(.6,.9),comb.fixed=TRUE, comb.random=TRUE,
       comb.r=T, comb.f=T,
```

```
       fontsize=7.8, digits=3,boxsize=0.8, digits.pval =
max(gs("digits.pval") - 2, 2),
       leftcols=c("studlab", "Year", "Comparison Model
(CM)","SampleSize"),
       leftlabs=c("Author", "Year", "CM Model Type", "Training and
Validation Set"),
       rightlabs=c( "AUROC","95% CI", "Weight (fixed)", "Weight
(random)"))
CM_model <-
read_excel("R:\\PrevMed\\Projects\\BCC\\Projects\\BarboiC_1416\\Analysis\\
Data\\CM model for analysis.xlsx")
APS_data <- CM_model[ which(CM_model$`Comparison Model (CM)` =='APS
III'|CM_model$`Comparison Model (CM)` =='APS III ICU mortality'),]

CM_APS<- metaprop( Numerator_decimal,SampleSize ,studlab=Author,
                   sm="PRAW", data = APS_data,method="Inverse",
method.tau="DL", method.ci = "NAsm")
CM_APSForest(CM_APS,  xlim = c(.6,.9),comb.fixed=TRUE, comb.random=TRUE,
       comb.r=T, comb.f=T,
       fontsize=7.8, digits=3,boxsize=0.8, digits.pval =
max(gs("digits.pval") - 2, 2),
       leftcols=c("studlab", "Year", "Comparison Model
(CM)","SampleSize"),
       leftlabs=c("Author", "Year", "CM Model Type", "Training and
Validation Set"),
       rightlabs=c( "AUROC","95% CI", "Weight (fixed)", "Weight
(random)"))
CM_model <-
read_excel("R:\\PrevMed\\Projects\\BCC\\Projects\\BarboiC_1416\\Analysis\\
Data\\CM model for analysis.xlsx")
qSOFA_data <- CM_model[ which(CM_model$`Comparison Model (CM)` =='qSOFA
12'|CM_model$`Comparison Model (CM)` =='qSOFA 24'|CM_model$`Comparison
Model (CM)` =='qSOFA48'|CM_model$`Comparison Model (CM)` =='qSOFA72'),]
CM_qSOFA<- metaprop( Numerator_decimal,SampleSize ,studlab=Author,
                     sm="PRAW", data = qSOFA_data,method="Inverse",
method.tau="DL", method.ci = "NAsm")
CM_qSOFA
forest(CM_qSOFA,  xlim = c(.65,.9),comb.fixed=TRUE, comb.random=TRUE,
       comb.r=T, comb.f=T,
       fontsize=7.8, digits=3,boxsize=0.8, digits.pval =
max(gs("digits.pval") - 2, 2),
       leftcols=c("studlab", "Year", "Comparison Model
(CM)","SampleSize"),
       leftlabs=c("Author", "Year", "CM Model Type", "Training and
Validation Set"),
      rightlabs=c( "AUROC","95% CI", "Weight (fixed)", "Weight (random)")
read_excel("R:\\PrevMed\\Projects\\BCC\\Projects\\BarboiC_1416\\Analysis\\
Data\\CM model for analysis.xlsx")
SAPS2_data <- CM_model[ which(CM_model$`Comparison Model (CM)` =='SAPS
II'),]
CM_SAPS2<- metaprop( Numerator_decimal,SampleSize ,studlab=Author,
                     sm="PRAW", data = SAPS2_data,method="Inverse",
method.tau="DL", method.ci = "NAsm")
CM_SAPS2
```

```
forest(CM_SAPS2,  xlim = c(.65,.85),comb.fixed=TRUE, comb.random=TRUE,
       comb.r=T, comb.f=T,
       fontsize=7.8, digits=3,boxsize=0.8, digits.pval =
max(gs("digits.pval") - 2, 2),
       leftcols=c("studlab", "Year", "Comparison Model
(CM)","SampleSize"),
       leftlabs=c("Author", "Year", "CM Model Type", "Training and
Validation Set"),
       rightlabs=c( "AUROC","95% CI", "Weight (fixed)", "Weight
(random)"))
CM_model <-
read_excel("R:\\PrevMed\\Projects\\BCC\\Projects\\BarboiC_1416\\Analysis\\
Data\\CM model for analysis.xlsx")
SOFA_data <- CM_model[ which(CM_model$`Comparison Model (CM)` =='SOFA'),]

CM_SOFA<- metaprop( Numerator_decimal,SampleSize ,studlab=Author,
                    sm="PRAW", data = SOFA_data,method="Inverse",
method.tau="DL", method.ci = "NAsm")
CM_SOFA
forest(CM_SOFA,  xlim = c(.55,.8),comb.fixed=TRUE, comb.random=TRUE,
       comb.r=T, comb.f=T,
       fontsize=7.8, digits=3,boxsize=0.8, digits.pval =
max(gs("digits.pval") - 2, 2),
       leftcols=c("studlab", "Year", "Comparison Model
(CM)","SampleSize"),
       leftlabs=c("Author", "Year", "CM Model Type", "Training and
Validation Set"),
       rightlabs=c( "AUROC","95% CI", "Weight (fixed)", "Weight
(random)"))
#Apache IV
CM_model <-
read_excel("R:\\PrevMed\\Projects\\BCC\\Projects\\BarboiC_1416\\Analysis\\
Data\\CM model for analysis.xlsx")
Apache4_data <- CM_model[ which(CM_model$`Comparison Model (CM)` =='APACHE
IV'),]
CM_Apache4<- metaprop( Numerator_decimal,SampleSize ,studlab=Author,
                       sm="PRAW", data = Apache4_data,method="Inverse",
method.tau="DL", method.ci = "NAsm")

CM_Apache4
forest(CM_Apache4,  xlim = c(.6,.9),comb.fixed=TRUE, comb.random=TRUE
comb.r=T, comb.f=T,
       fontsize=7.8, digits=3,boxsize=0.8, digits.pval =
max(gs("digits.pval") - 2, 2),
       leftcols=c("studlab", "Year", "Comparison Model
(CM)","SampleSize"),
       leftlabs=c("Author", "Year", "CM Model Type", "Training and
Validation Set"),
       rightlabs=c( "AUROC","95% CI", "Weight (fixed)", "Weight
(random)"))
```
