# Supplementary material for: Comparison of Severity of Illness Scores and Artificial Intelligence Models That Are Predictive of Intensive Care Unit Mortality: Meta-analysis and Review of the Literature
Source: JMIR Med Inform. 2022 May 31;10(5):e35293. doi: 10.2196/35293 (PMC9198821; doi:10.2196/35293)
Supplement: Multimedia Appendix 2 [file medinform_v10i5e35293_app2.pdf]

Multimedia appendix 2

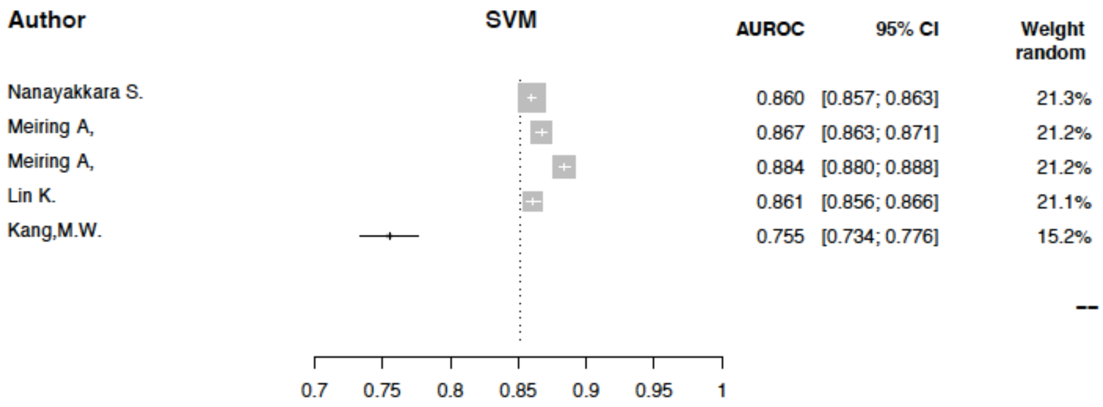

Figure 8. Support Vector Machine based models forest plot Heterogeneity: $I^2=98\%$, $\tau^2=0.0003$, $P<.01$

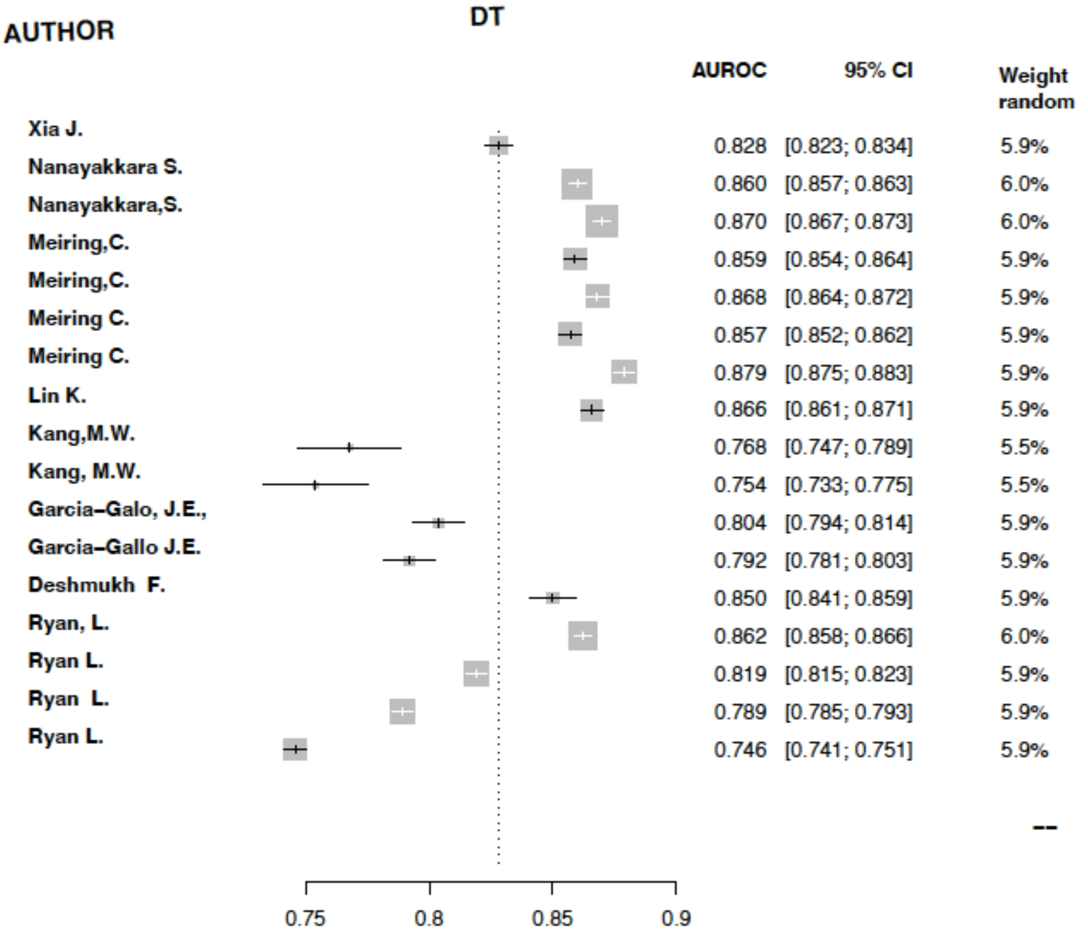

Figure 9. Decision Trees based models forest plot Heterogeneity: $I^2=100\%$, $\tau^2= 0.0015$, $P=0$

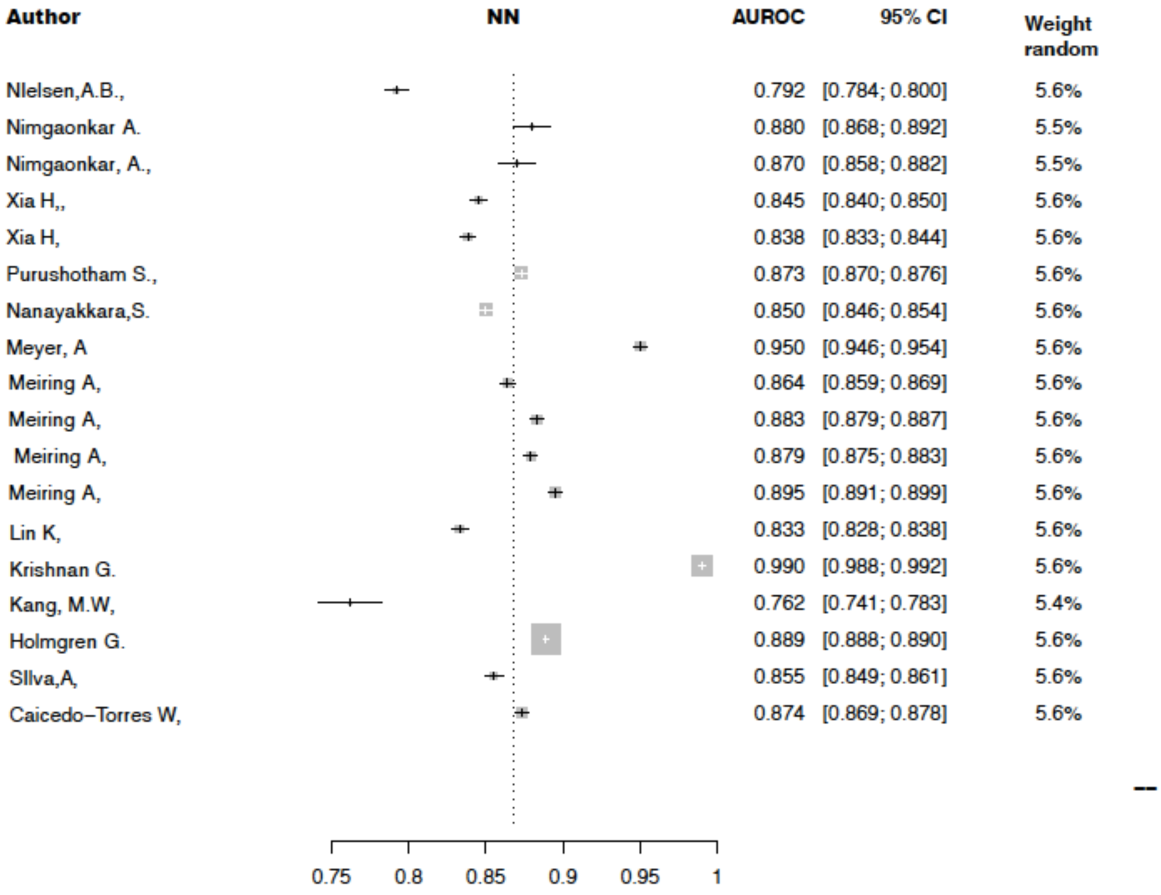

Figure 10. Neural Network based models forest plot Heterogeneity: I^2=100%, τ^2=0.0029, P=0

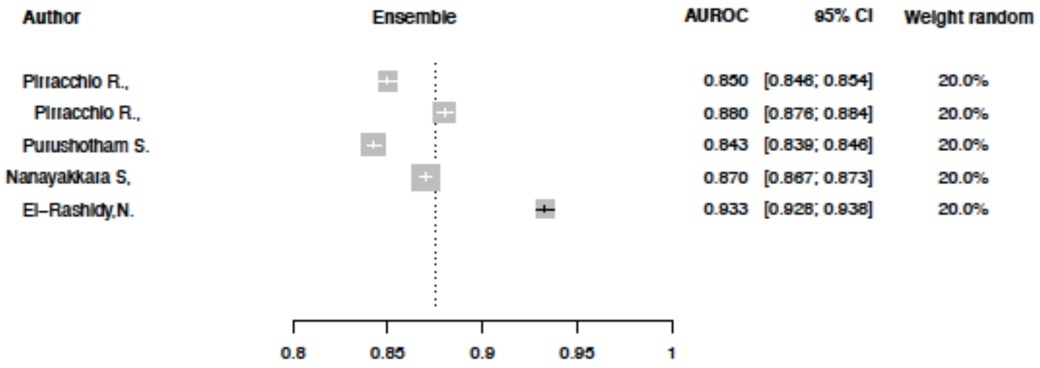

Figure 11. Ensemble based models forest plot Heterogeneity: I^2 =100%, τ^2=0.0010, P< .01
